# Supplementary material for: Effectiveness and Safety of Ayurvedic Medicines in Type 2 Diabetes Mellitus Management: A Systematic Review and Meta-Analysis
Source: Front Pharmacol. 2022 Jun 8;13:821810. doi: 10.3389/fphar.2022.821810 (PMC9213670; doi:10.3389/fphar.2022.821810)
Supplement: Supplementary file 1 [file DataSheet1.zip › Supplementary Material/Forest and Funnel Plots/Sesamum indicum/HbA1c.pdf]

Sesamum indicum - HbA1c

Study	Intervention N	Intervention Mean	Intervention SD	Comparator N	Comparator Mean	Comparator SD	Mean Diff. with 95% CI	Weight (%)
Shahi 2017	22	7.51	1.14	22	7.83	1.85	-0.32 [-1.23, 0.59]	49.07
Aslam 2018	23	6.96	1.25	23	8.02	1.77	-1.06 [-1.95, -0.17]	50.93
**Overall**							-0.70 [-1.42, 0.03]	

Heterogeneity: $\tau^2 = 0.06$, $I^2 = 23.46\%$, $H^2 = 1.31$

Test of $\theta_i = \theta_j$: Q(1) = 1.31, p = 0.25

Test of $\theta = 0$: z = -1.88, p = 0.06

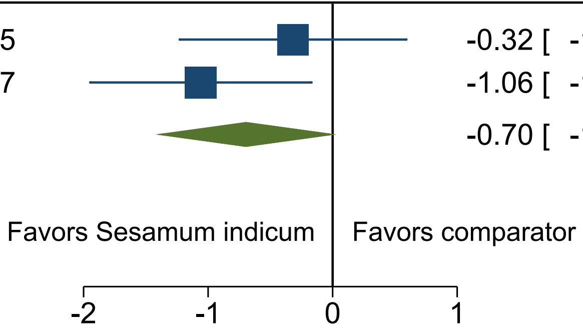

Random-effects REML model
